# Effect of Refined Perioperative Nursing on the Efficacy of Noninvasive Ventilation in Elderly Patients with Lung Cancer and Respiratory Failure

**DOI:** 10.1155/2022/4711935

**Published:** 2022-06-15

**Authors:** Li Huang, Chun Feng

**Affiliations:** ^1^Department of Geriatric Intensive Care Unit Nursing, Sichuan Provincial People's Hospital, University of Electronic Science and Technology of China, Chengdu 610072, China; ^2^Sichuan Translational Medicine Research Hospital, Chinese Academy of Sciences, Chengdu 610072, China

## Abstract

NIV (noninvasive ventilation) is becoming more popular as a first-line treatment for older patients with lung cancer who are experiencing acute respiratory failure. In the ICU, older age is linked to worse results with mechanical breathing. When dealing with severely sick patients, noninvasive ventilation is beneficial. Due to the risk of NIV failure and the higher mortality induced by delayed intubation, it is difficult to apply to older patients, especially those with lung cancer and respiratory insufficiency. As a result, for a successful outcome, nurse interventions should be provided to patients during noninvasive ventilation. This paper proposes the application of integrated perioperative nursing models on the elderly patients with lung cancer and respiratory failure. We have applied three nursing models: peer support nursing model, multidisciplinary cooperative nursing model, and transcultural nursing theory. The effect of the proposed nursing model on the efficacy of NIV is evaluated using the Logical Decision Tree Regression (LDTR) model. It is optimized using Iterative Fruit Fly Optimization Algorithm (IFOA). The performance of the suggested system is analysed, and it is observed that the patients showed better surgical outcomes when provided with the integrated nursing models.

## 1. Introduction

**Figure 1:**
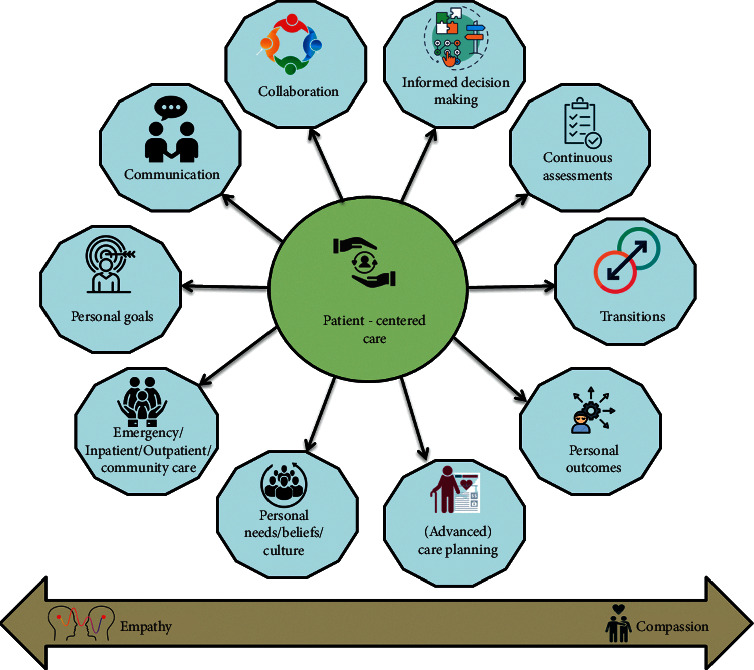
Patient-centred care flow.

Lung cancer caused by smoking is one of the most dangerous malignancies, with morbidity and mortality rising at the fastest pace in the world, especially among the elderly. As shown in Figure 1, the large rise in the number of elderly individuals in recent years has made lung cancer prevention and control more important than ever before. Patients' treatment is being closely monitored, as indicated in Figure 1. Around 80% of all occurrences of lung cancer are small cell, making it the most common kind. It is a complex process that includes a variety of risk factors in the development of lung cancer. Several risk factors for lung cancer, including smoking [1], second-hand smoke [2], high levels of air pollution exposure [3], and drinking water with a high amount of arsenic [4], may increase the likelihood of the disease developing. The link between these risk variables and the incidence of lung cancer is a pressing scientific question that requires immediate attention.

Increasing population-based survival for specific malignancies in high-income nations has been shown to be beneficial when early diagnosis, screening, and treatment are used in conjunction with one another. Many types of research on lung cancer screening have been conducted in the previous few years. An American study called the National Lung Screening Trial (NLST) examined whether low-dose computed tomography (CT) may reduce lung cancer mortality. A yearly screening for persons 56 to 81 years old who have a 30-pack-year smoking history, are presently smoking, or have quit smoking within the previous fifteen years was recommended by the US Preventive Services Task Force in 2010. Estimates for each of six smoking status classes were created using multilevel small-area estimation mixed methods. With each passing year, unhealthy lifestyle choices and environmental pollutants are contributing to a rise in the incidence and death of NSCLC non-small-cell lung cancer. Non-small-cell lung cancer, which accounts for more than 81 percent of all lung cancer cases, is associated with a high rate of morbidity and death around the globe. Debilitating illnesses come in a wide variety of forms, and each has a unique effect on the way people live their lives. According to several reports, cancer discomfort is often accompanied by feelings of worry or sadness. The worldwide burden of lung cancer is continually growing, according to several studies, due to an increase in cancer-causing behaviours. Many people suffer from excruciating discomfort for which there is now no effective treatment. Integrated perioperative care, a kind of long-term care that enhances the quality of a patient's life while also reducing the need of medical services, has been implemented in a number of countries throughout the globe, with a particular emphasis on terminal cancer treatment. Patient symptoms, as well as family satisfaction with treatment, obtaining aid with decision-making, enhancing patients' subjective well-being, and communicating with healthcare professionals are all goals of integrated perioperative care. Because it must cater to a wide range of requirements, integrated perioperative care must be thorough, interdisciplinary, and patient- or family-centred in its approach. To address these drawbacks, we recommend that integrated perioperative nursing models be used on older patients with lung cancer and respiratory failure in order to improve outcomes. Three nursing models were used in this study: the peer support nursing model, the interdisciplinary cooperative nursing model, and the transcultural nursing theory are all examples of nursing models. The remaining part of this article organized as follows: Section 2 related work deeply analysis pervious article and found out problems.  Section 3 presents materials and methods of suggested flow.  Section 4 presents proposed results and discussed their parameter.  Section 5 presents conclusion of this article.

## 2. Related Work

Researchers [5] examined the postoperative outcomes of lung cancer resections done on patients 60 and older during January 2013 and December 2014 using the Dutch Lung Surgery Audit database. A multivariate analysis looked at postoperative complications and surgical mortality. Long-term outcomes and prognostic indicators for elderly patients with acute respiratory failure (ARF) following invasive mechanical ventilation are lacking due to a lack of study (IMV) [6]. There has been an increase in lung cancer incidence among the older populations who are otherwise healthy, with intact pulmonary function and no other underlying lung conditions [7]. PPCs (postoperative pulmonary complications) are common in older individuals with non-small-cell lung cancer (NSCLC), and this research aims to identify the best appropriate candidates for surgical resection in this group of patients [8]. NIV is increasingly being used as a palliative treatment for respiratory insufficiency and dyspnoea. Noninvasive ventilation is referred to as NIV. An investigation on the effects of NIV on elderly patients is the subject of this narrative review [9]. It was determined if NIV was acceptable for older patients with pneumonia in this prospective observational study, which also looked for factors that indicated NIV failure on their own, and then established an ideal model for predicting NIV failure in this particular group of patients. In this research, a low PaCO_2_ level, a rapid heart rate, and pneumonia were all shown to be significant predictive factors of NIV failure [10]. The Danish Lung Cancer Screening Trial screening arm participants (*n* = 1990) with relevant baseline CT scan data were included in this cohort analysis. In some cases, patients were monitored for up to 12 years after their baseline scans were screened for ILA. Cox proportional hazards models were used to estimate the participants' disease-specific morbidity and healthcare use using data from the Danish National Health Registries. Cox proportional hazard models were used to analyse all hospital admissions, primary healthcare visits, and medications [11]. Accordingly, rather than providing an accurate estimate of the impact of PM_2.5_ on lung cancer risk, this study is demonstrating that PH model calculations are essentially inaccurate. This study found that the risk ratio (HR) for lung cancer associated with pack-years of smoking was significantly influenced by age when compared to a best-fitting model that took into account all three variables: time since stopping, duration, and pack-years. This study's purpose was to see whether there were any changes in the quantity and kinds of symptom clusters based on whether or not the incidence rates or symptom severity ratings were utilized to generate the clusters in these individuals [12]. Cancer caused by smoking has the highest death rate of any malignant tumour in older people. Lung cancer control and prevention have become more significant in recent years due to the growing growth of the senior population. To make matters worse, the pathophysiology of lung cancer is very complicated and includes several risk factors [13]. As part of this study, researchers applied a deep learning technology in order to identify and quantify major risk variables for lung cancer occurrence in older people [14]. The functional status of children was assessed during their mechanical assistance for acute respiratory failure and again six months after they were discharged as part of the RESTORE Randomized Evaluation of Sedation Titration for Respiratory Failure project. The Infant and Toddler Quality of Life Questionnaire-97 and the Pediatric Quality of Life Inventory were used to assess HRQL in the children. Screening for lung cancer using an individual's own risk factors rather than age and smoking history is becoming more popular. Tests were conducted on the capacity of nine previously developed risk models to identify those at high risk of acquiring or dying of lung cancer. All models took into account factors such as current smoking status, length of smoking, number of cigarettes smoked per day, number of pack-years smoked, and the amount of time since a person last smoked [15]. There were 1461 lung cancer and 954 colorectal cancer patients recruited in this study. In a multivariate logistic regression, it was shown that living less than 50 meters from a major road and smoking were both significantly associated with an increased risk of lung cancer. Based on findings from the GWLR model, the correlation between proximity to a major roadway and lung cancer risk varied depending on where you resided [16]. We did a pooled analysis using data from eight studies submitted to the International Lung Cancer Consortium (4,386 cases and 4,177 controls). Reproductive and menstrual factors were correlated with lung cancer using multivariable unconditionally logistic regression in a large sample. Menopausal status, smoking habits, and histopathology were all taken into consideration while doing subgroup analysis. Peri- and postmenopausal women had a higher incidence of lung cancer than premenopausal women, but no strong evidence supported a link between menopause and lung cancer [17]. The study's goal was to find out whether the fear of lung cancer, the perceived risk, and the synergistic risk of smoking in the home were linked, while also controlling for factors including sociodemographic characteristics, family background of lung disease, and wellness self-concept [18]. The assumption is that people who smoke inside have higher lung cancer dread, risk involved, and synergy risk scores than nonsmokers. An examination of NLST data that included 22229 individuals indicated that the PLCOm2012 and National Lung Screening Trial findings (PLCO2012 results) improved the accuracy of the prediction of future lung cancer by a substantial margin compared to a model that omitted the results (a model that did not incorporate results) [19]. With the help of the CISNET project, an LC screening microsimulation model was constructed. Data from the NLST (National Lung Screening Trial) and PLCO were used to calibrate and validate the model, respectively. The outcomes of the USPSTF-recommended lifelong screening programme might be evaluated at the population level by varying the degree of screening compliance. With the help of the CISNET project, an LC screening microsimulation model was constructed. Both the National Lung Screening Trial and Ovarian Cancer Screening Trial used to calibrate and system validated for lung and colorectal, respectively. We were able to examine the outcomes of the USPSTF's lifetime screening programme at the population level by modifying the degree of screening compliance [20]. There is a wide range of treatment options and outcomes when it comes to lung cancer, which is Europe's most common cancer mortality cause [21]. A multidisciplinary core team (MDT) and an extended team of health professionals are required for the care of lung cancer patients exclusively at lung cancer units or centres, as detailed in this paper.

## 3. Material and Methods

**Figure 2:**
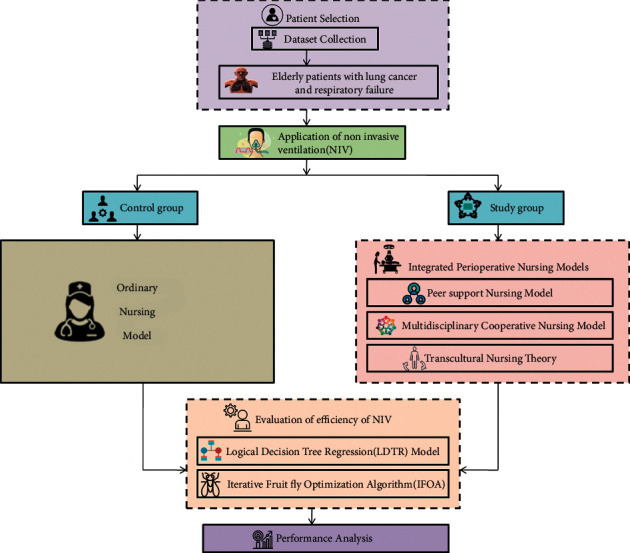
Overall proposed flow.

In this section, we suggest the implementation of integrated perioperative nursing models on the elderly patients with lung cancer and respiratory failure, overall illustrated in Figure 2. We have utilised three nursing models: multidisciplinary cooperative nursing model, peer support nursing model, and transcultural nursing theory. The influence of the suggested nursing model on the effectiveness of NIV is examined using the Logical Decision Tree Regression (LDTR) model. To assess the risk factor effectively by utilizing Iterative Fruit Fly Optimization Algorithm (IFOA).

### 3.1. Data Set

A prospective trial of 104 patients with lung cancer and respiratory failure admitted to Sichuan Provincial People's Hospital was conducted over the course of three years [March 2018 to March 2021]. The patients were assigned to two groups at random: control (*d* = 52) or study (*d* = 52). In the control group, the ordinary nursing approach was applied, and in study group, the peer support nursing, multidisciplinary cooperative nursing, and transcultural nursing models were applied (Table 1).

### 3.2. Application of Noninvasive Ventilation

The use of noninvasive ventilation (NIV) in the therapy of lung cancer and respiratory failure is growing in popularity, owing to a reduction in the number of (potential) complications associated with endotracheal intubation and other medical interventions. Such an effect might help to reduce mortality and the length of time a patient stays in the hospital. NIV may be used to offer step-up care for patients who have failed standard medical therapy for lung cancer and respiratory failure when used in combination with a step-care plan. Depending on their requirements, continuous positive airway pressure (CPAP) and bilevel positive airway pressure (BPP) are two options for mechanical ventilation patients. The favourable findings of a pilot trial in 10 people with symptomatic hypercapnic or hypoxemic respiratory failure have piqued the curiosity of researchers in the use of NIV to treat acute lung cancer and acute respiratory failure. Numerous case studies revealed a favourable impact of NIV in acute lung cancer and respiratory failure of various aetiologies, prospective randomized trial studies investigating the role of NIV in cancer of the lung and respiratory failure. Since there have been an additional eight randomized controlled trials on the effect of NIV in lung cancer and respiratory failure published to date, a meta-analysis update was performed with the inclusion of the most recently published studies in this field. The main purpose of this update was to see whether NIV may reduce mortality in patients with lung cancer and respiratory failure, as well as lung cancer linked to COPD exacerbations and other non-COPD parenchymal processes. Secondary outcomes included the requirement for mechanical ventilation, length of stay in the hospital, and the occurrence of complications. It was also able to illustrate the application of numerous recently found statistical approaches to meta-analysis, which were also shown. We separated the data set into two groups to test the efficacy of the NIV: the control group and the study group.

### 3.3. Nursing Model

#### 3.3.1. Control Group

- Ordinary nursing model: NIV was followed by standard treatment. The cause of the patient's illness was determined swiftly, and the patient's condition was quickly examined. The purpose of first aid was to open the airway as fast as possible, offer medication as recommended by the doctor, and monitor the patient's condition on a frequent basis after the first aid was administered in order to maintain the airway clean and encourage respiratory recovery.

#### 3.3.2. Study Group (Integrated Perioperative Nursing Model)

- Peer support nursing model: for those who are physically and psychologically well, peer support nursing is a kind of nursing practice that aids those who are rehabilitating. Face-to-face and virtual platforms are used in various countries. Shared experiences and professional lives have been demonstrated to lessen patients' feelings of loneliness and anxiety, therefore increasing their quality of life throughout the pre- and postsurgical period. Peer support nursing was utilized to organize and guide volunteers and aid patients in the preoperative and postoperative phases; the meetings were conducted once a week and included themes such as emotional support and rehabilitation experience. After a two-month intervention, patients with lung cancer and respiratory failure saw improvements in their physical and mental health, as well as fewer surgical complications. However, the intervention also resulted in considerable savings in healthcare personnel. Furthermore, the peer support model necessitates the establishment of standardized lung cancer volunteer institutions by a hospital or organization, in addition to the usual input of medical personnel for training; they may only enter hospitals to help patients with the same ailment when they have fulfilled training criteria.
- Multidisciplinary cooperative nursing model: it has recently emerged as an essential medical diagnostic model: the multidisciplinary team (MDT), wherein diagnostic and therapeutic processes are optimized, medical resources are wisely allocated, and diagnostic and therapeutic results are improved via multidisciplinary partnership. In many circumstances, the MDT includes not only surgeons and doctors but also lung care specialists, physiotherapists, peer support specialists, consultants, and psychologists as members of the group. This paradigm, which takes a multidisciplinary approach to address the needs of lung patients and families, has long been recognized as valuable by doctors, nurses, allied health workers, and even patients. For the comparison group, the hospital's traditional nursing paradigm was employed, whereas for the observation group, an MDT was set up. An experienced lung surgeon served as liaison between the team's first-level members, which included a nurse specialist, bed nurse, and nutrition physicians among the second-level team members, psychological rehabilitation, plastic surgery, imaging, pathology, and conventional Chinese drug. For as long as the MDT remained active, all first-level members were required to attend two weekly consultation sessions, with second-level members able to attend if circumstances warranted.
- Transcultural nursing theory: Madeleine Leininger, a nursing scholar from the USA, was the first to develop the transcultural nursing theory, which was then presented in China at 1995. Sunrise Nursing Mode (SNM) is the foundation of transcultural nursing. Nurses are seen by SNM as delivering tailored care to patients from a variety of ethnic and cultural backgrounds, rather than a one-size-fits-all paradigm. Patients' views, educational backgrounds, languages, and personal economic situation were examined in the first level. Second, we looked at the health of the patients and their carers, as well as how much money they had spent on medical treatment. Level 3 examined patients' medical and nursing care requirements. Specific nurse interventions were then carried out based on the findings of an evaluation of the patient's language barriers in the areas of mental health, life care, and postoperative care. Many studies have shown that a transcultural nursing model helps to prevent cultural coercion and culture shock due to cultural differences, improves the quality of nursing care provided to international patients, fosters a more humane work environment, and adheres to the global trend of global integration.

### 3.4. Logical Decision Tree Regression

This study employs Logical Decision Tree Regression, as well as a tool that generates polynomial toy data, and divides it into training and validation sets at random. The class Logical Decision Tree Regression is described in this paper. We begin by assuming that our hypothesis *h*(*x*) is a linear mixture of a few different basis functions

(1) $$\begin{array}{c} h\left(v\right)=\beta^{M}\mu \left(v\right). \end{array}$$

We should calculate *β*. We suppose that the difference between some targeted variable Γ and a quantity of deterministic function is distorted by additive noise, just as with ordinary regression.

(2) $$\begin{array}{c} \Gamma =\beta^{M}\mu \left(v\right)+\sigma . \end{array}$$

Maximum may be used to demonstrate the Gaussian assumption on Γ and on the parameters. We may acquire the optimum vector values using a posteriori estimation by *β* minimizing the subsequent cost function task.

(3) $$\begin{array}{c} C\left(\beta \right)=\sum_{i=1}^{N}\Gamma_{i}-\beta^{M}\mu {\left(v_{i}\right)}^{2}+\frac{\lambda }{2}\beta^{M}\beta . \end{array}$$

It is not difficult to demonstrate that the quantity of *β* that minimizes equation (3) is provided by

(4) $$\begin{array}{c} \beta =\left[I\lambda {\left(\sum_{i=1}^{N}\mu \left(v_{i}\right)\mu \left(v_{i}\right)\right)}^{-1}\left[\sum_{i=1}^{N}\Gamma \mu \left(v_{i}\right)\right]\right]. \end{array}$$

We may wish to describe Equation (1) in terms of Logical if the dimension of *μ*(*v*) is quite big and for a variety of other reasons. Using Lagrange multipliers, we proved that Equation (1) may be stated in the form

(5) $$\begin{array}{c} h\left(v\right)=l{\left(v\right)}^{M}{\left(L+\lambda I\right)}^{-1}\Gamma , \end{array}$$

where the Logical is given by

(6) $$\begin{array}{c} L\left(v,u\right)=\mu^{M}\left(v\right)\mu \left(u\right). \end{array}$$

The predictor variables from the learning algorithm form the matrix *L*, which is a gram matrix with component (*L*)_*ij*_=*l*(*v*_*i*_, *v*_*j*_). *k*(*v*) is a vector whose *i*-th component has the form as follows:

(7) $$\begin{array}{c} \left(l\left(v\right)\right)i=\mu^{M}\left(v_{i}\right)\mu^{M}\left(v_{j}\right). \end{array}$$

The *i*-th component sample is denoted *v*_*i*_. There are a few conditions that must be followed in order to change anything in a legitimate Logical. Finally, the data were successfully analysed using a nursing model for both the control and study groups.

### 3.5. Iterative Fruit Fly Optimization Algorithm

Programming that mimics fruit fly foraging behaviour is called the Iterative Fruit Fly Optimization Algorithm (IFOA). Using the Iterative Fruit Fly Optimization technique, global optimization may be improved in an incremental manner. In the beginning, it all began with a study of Iterative Fruit Fly optimization food gathering behaviours. Fruit fly is a great food hunter with excellent eyesight and apheresis. In the beginning, it smells a range of fragrances floating about and goes to the suitable position to discover a food source. It is able to identify food or move to a specified spot when it gets close enough to the food, thanks to its excellent vision. The FOA iteratively seeks for the optima, which represents food sources, and the approach of foraging is repeated by iteratively looking for the optima. The IFFOA method is an upgraded version of the Iterative Fruit Fly Optimization algorithm. It outperforms the fruit fly algorithm in terms of performance.

### 3.6. Algorithm for IFFOA

Data: Position is blocked by low variance at first.
Result: Blocks in the best location.
Phase 1: Initialization of parameters: the total evolution number and the location of low variance blocks are the two primary parameters of the IFFOA. The low variance block location is represented in our recommended method by the Iterative Fruit Fly Optimization Algorithm. Place low variance blocks in a random place at first (*R*_*x*_ axis and *R*_*y*_ axis).
Phase 2: the Iterative Fruit Fly Optimization approach is used to improve on the standard fruit fly algorithm. The current agent and its opposing agent are examined concurrently in OBL opposition based learning to acquire a better estimate for the present accomplishing. It is assumed that an opposing agent solution has a greater likelihood than a random agent solution of being closer to the global optimum solution. Components entirely describe the placements of the opposing variance blocks.
  (8) $$\begin{array}{c} PR_{s}=\left[PR_{s}^{1},PR_{s}^{2},\ldots ,PR_{s}^{d}\right], \end{array}$$
where *PR*_*s*_=Low_*s*_+*Ur*_*s*_ − *R*_*s*_ with *PR*_*s*_ ∈ [Low_*s*_, *Ur*_*s*_ ] is the position of *s*^th^ low variance blocks *PR*_*s*_ in the *d*-th dimension of oppositional blocks.
Phase 3: exploring with an unlimited route and a low variety of service block is selected, where *R*_*s*_ denotes the *s*^th^ variety of service block position.
  (9) $$\begin{array}{c} R_{s}\left(v,u\right)={\left({\text{RV}}_{s},{\text{RU}}_{s}\right)}^{M},\\ {\text{RV}}_{s}={\text{RV}}_{-}\text{axis}+\text{Rv},\\ {\text{RU}}_{s}={\text{RU}}_{-}\text{axis}+\text{Rv}. \end{array}$$
Here, Rv denotes random variable.
Phase 4: position assessment of the technique suggested
  (10) $$\begin{array}{c} {\text{FN}}_{s}=\text{KL}. \end{array}$$
Step 5: in the fitness value, we substitute the location of variety of service blocks
  (11) $$\begin{array}{c} \text{Best block}=\text{function}\left(\text{Min }{\text{FN}}_{s}\right). \end{array}$$
Phase 6: find the best low-variance block placements.
  (12) $$\begin{array}{c} \left[\text{Eb},\text{Es}\right]=\text{M}\_\text{error}. \end{array}$$
Here, Eb represents excellent box, Es represents excellent selection, and M_error represents Min error.
Phase 7: the Iterative Fruit Fly Optimization method will use visualization to fluttering in the path of the optimal location of variety of service block value and *v*, *u* coordinate.
  (13) $$\begin{array}{c} Sb=M\_error, \end{array}$$
where Sb represented selected block
  (14) $$\begin{array}{c} {\text{RV}}_{-}\text{axis}=\text{RV}\left(ei\right),\\ {\text{RU}}_{-}\text{axis}=\text{RU}\left(ei\right), \end{array}$$
where *ei* represents excellent index
Phase 8: decide whether the current location of variety of service blocks is better than the previous position of variety of service blocks by using successive optimization to repeat the execution of phases three to six. If yes, carry out the assignment.

The Iterative Fruit Fly Optimization Algorithm analyses factors such as quality of life, negative mood, self-efficacy, and lung function.

## 4. Result and Discussion

In this section, we analyses the following factors: lung function, negative mood, quality of life, and self-efficacy. The control group and the study group are compared and evaluated.

### 4.1. Lung Function

**Figure 3:**
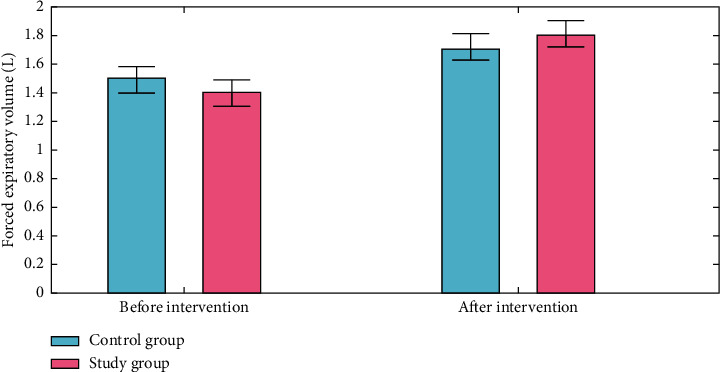
Forced Expiratory volume analysis for control group and study group.

**Figure 4:**
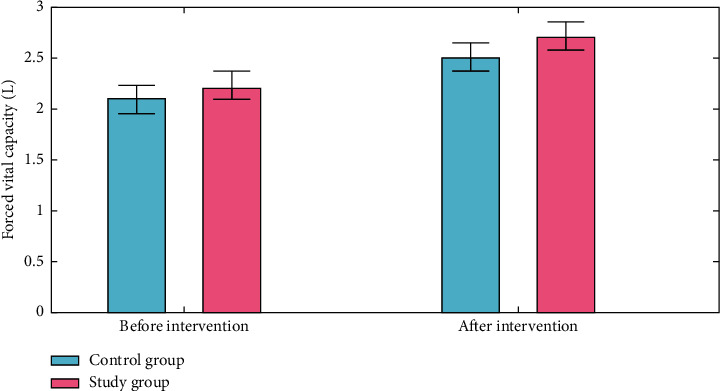
Forced vital capacity analysis for the control group and the study group.

**Figure 5:**
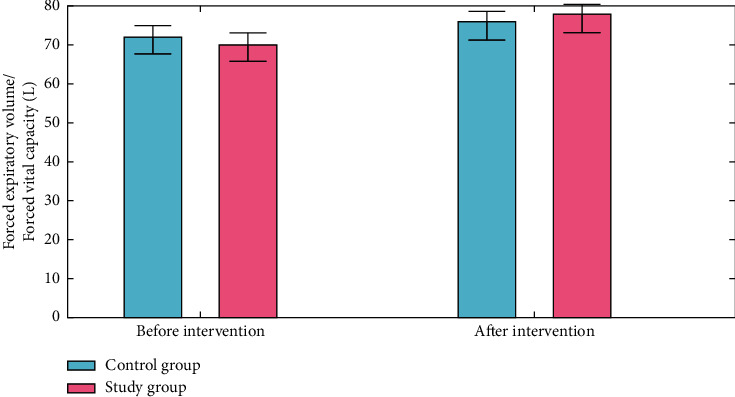
Forced vital capacity analysis for the control group and the study group.

An “forced expiratory volume (FEV)” test measures the amount of air a person can expel when they forced breathe. The amount of air that is pushed out can be counted during the first, second, and/or third seconds of the forced breath, as well. Forced vital capacity (FVC) is the overall volume of air that was breathed during the FEV test. The first forced expiratory volume, forced vital capacity, and forced expiratory volume/forced vital capacity (the proportion of the forced vital capacity to the initial forced expiratory volume) of the two groups were all measured using the spirometer. Both groups had higher forced expiratory volume, forced vital capacity, and forced expiratory volume/forced vital capacity than before intervention, and the experimental group had higher forced expiratory volume/forced vital capacity and forced expiratory volume/forced vital capacity than the control group (*P* < 0.050) (Figures 3–5).

### 4.2. Negative Mood

**Figure 6:**
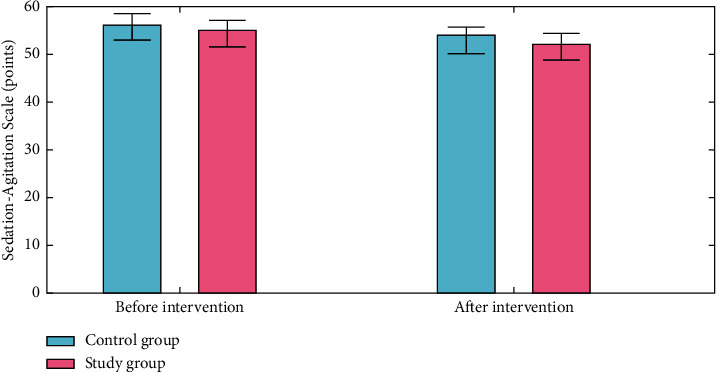
Sedation Agitation Scale analysis for the control group and the study group.

**Figure 7:**
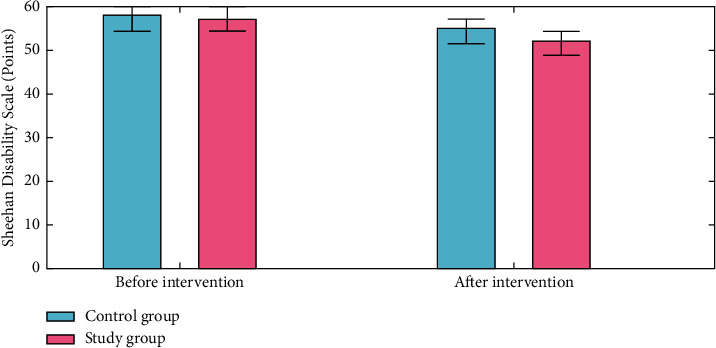
Sheehan Disability Scale analysis for the control group and the study group.

The Zung Self-rating Anxiety Scale (SAS) and the Zung Self-rating Depression Scale (SDS) were used to measure anxiety and depression. The reliability and validity of both measures are excellent. There were 52 items on the Self-Rating Anxiety Scale (SAS) and the Self-Rating Depression Scale (SDS). A Self-Rating Anxiety Scale score of zero denotes no anxiety, 51–58 suggest mild anxiety, 61–68 represent moderated anxiety, and >68 points imply severe anxiety. More severe anxiety symptoms are indicated by a higher total score. As a result, a standard score of more than 50 indicates the existence of anxiety. No depression is indicated by an SDS score of less than 52 points, mild depression by 52–61 points, moderate depression by 62–71 points, and severe depression by >72 points. A higher score suggests that you are experiencing more negative feelings. To qualify as depressed, a standard score of 53 or above on the SDS is required. SAS was 0.803, and SDS was 0.806. We found that both groups had a decrease in their SAS and SDS scores as a result of intervention, with the study group having lower SAS and SDS scores than those in the control group (*P* < 0.050) as shown in Figures 6 and 7. The formula used to normalize the raw score is as follows:

(15) $$\begin{array}{c} \text{Standard score}=\mathrm{int}\left(1.25\times \text{raw score}\right). \end{array}$$

### 4.3. General Self-Efficacy Scale Scores

**Figure 8:**
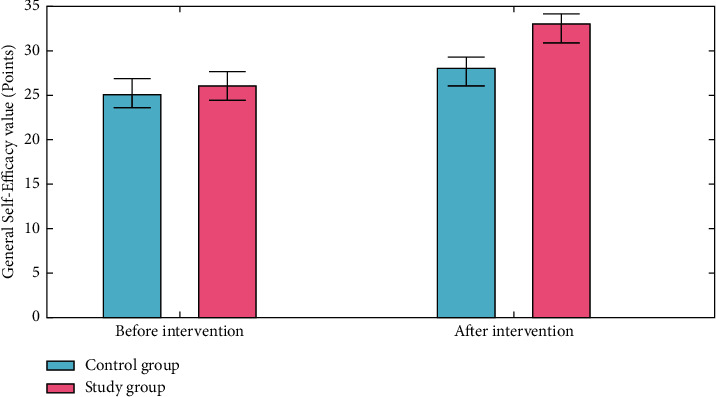
General Self-Efficacy Scale analysis for the control group and the study group.

GSE (General Self-Efficacy Scale) are associated with emotion, optimism, and job satisfaction. The final score is evaluated by finding the sum of the all items. Totally, 102 patients are analysed to find out the General Self-Efficacy Scale scores. The overall score ranged from 10 to 40 points, with poor efficiency receiving 10 to 20 points, moderate efficiency 21 to 30 points, and high efficiency 31 to 40 points. Self-efficacy is measured by a score. A higher score implies more self-efficacy. The General Self-Efficacy Scale coefficient was 0.859. As demonstrated in Figure 8, the study group's General Self-Efficacy Scale scores were significantly higher than those of the control group (*P* < 0.050) after treatment.

### 4.4. Quality of Life

**Figure 9:**
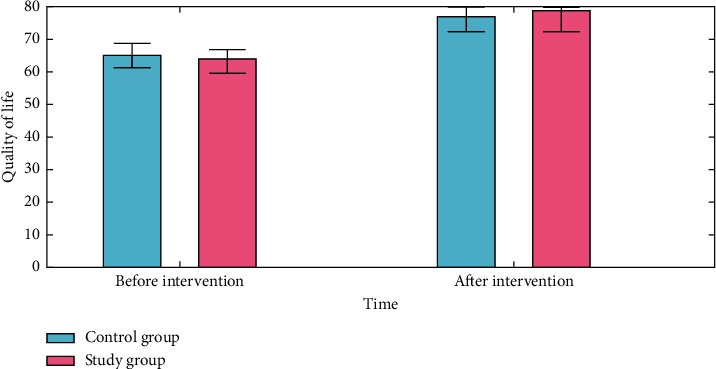
Quality-of-life analysis for the control group and the study group.

There was a statistically significant difference in the quality-of-life scores between the study and control groups (*P* < 0.050). Figure 8 shows the situation. It contained 102 items in total, comprising four dimensions such as physical function, emotional activity, general health, and an extra health change, with each dimension's score ranging from 0 to 100 points. Each dimension's average score was computed. A higher score denotes a greater standard of living (Figure 9).

## 5. Conclusion

When compared to the control and study groups, patients who received an early integrated peri-operative nursing approach had considerably better quality of life, negative mood, and lung function. In this paper, nursing model is divided into two groups: one is the control group and another one is the study group; the ordinary nursing model was used in the control group; and the study group used the peer support nursing, multidisciplinary cooperative nursing, and transcultural nursing models. This strategy supports patients in maintaining a good state of mind by presenting the appropriate psychological treatment and boosting nurse-patient contact. To a certain degree, music education and sports training may lessen the clinical symptoms of patients with lung cancer and respiratory failure as well as enhancement health and cognition. Patients' general well-being has improved as a result of significant shifts in perspective, treatment coordination, and day-to-day habits.

## Figures and Tables

**Table 1:** Data set description.

Data		Control group (*d* = 52)	Study group (*d* = 52)	*X* ^2^	*P*
Age (years)	<64	24 (45.17)	25 (48.02)	0.156	0.695
	>64	28 (54.83)	27 (51.99)		

Gender	Male	27 (53.75)	26 (50)	0.155	0.696
	Female	25 (46.25)	26 (50)		

Type	Type 1	31 (60.54)	37 (44.67)	1.076	0.298
	Type 2	21 (39.46)	15 (57.59)		

Cause of disease	Critical asthma	12 (22.15)	10 (20.99)	1.187	0.988
	Respiratory affliction syndrome	7 (13.47)	7 (13.47)		
	Lung cancer	25 (45.41)	23 (43.99)		
	Sleep apnoea syndrome	8 (18.97)	12 (21.55)		

Smoking history	Present	24 (47.58)	23 (43.52)	0.068	0.815
	Absent	28 (52.42)	29 (56.48)		

## Data Availability

The analysed data sets generated during the study are available from the corresponding author on reasonable request.
